# A parahippocampal-sensory Bayesian vicious circle generates pain or tinnitus: a source-localized EEG study

**DOI:** 10.1093/braincomms/fcad132

**Published:** 2023-04-20

**Authors:** Dirk De Ridder, Karl Friston, William Sedley, Sven Vanneste

**Affiliations:** Unit of Neurosurgery, Department of Surgical Sciences, Dunedin School of Medicine, University of Otago, Dunedin 9016, New Zealand; Wellcome Trust Centre for Neuroimaging, University College London, London WC1N 3AR, UK; Translational and Clinical Research Institute, Newcastle University, Newcastle upon Tyne NE1 7RU, UK; School of Psychology, Trinity College Dublin, Dublin D02 PN40, Ireland; Global Brain Health Institute & Institute of Neuroscience, Trinity College Dublin, Dublin D02 PN40, Ireland

**Keywords:** pain, tinnitus, theta, gamma, parahippocampal

## Abstract

Pain and tinnitus share common pathophysiological mechanisms, clinical features, and treatment approaches. A source-localized resting-state EEG study was conducted in 150 participants: 50 healthy controls, 50 pain, and 50 tinnitus patients. Resting-state activity as well as functional and effective connectivity was computed in source space. Pain and tinnitus were characterized by increased theta activity in the pregenual anterior cingulate cortex, extending to the lateral prefrontal cortex and medial anterior temporal lobe. Gamma-band activity was increased in both auditory and somatosensory cortex, irrespective of the pathology, and extended to the dorsal anterior cingulate cortex and parahippocampus. Functional and effective connectivity were largely similar in pain and tinnitus, except for a parahippocampal-sensory loop that distinguished pain from tinnitus. In tinnitus, the effective connectivity between parahippocampus and auditory cortex is bidirectional, whereas the effective connectivity between parahippocampus and somatosensory cortex is unidirectional. In pain, the parahippocampal-somatosensory cortex is bidirectional, but parahippocampal auditory cortex unidirectional. These modality-specific loops exhibited theta–gamma nesting. Applying a Bayesian brain model of brain functioning, these findings suggest that the phenomenological difference between auditory and somatosensory phantom percepts result from a vicious circle of belief updating in the context of missing sensory information. This finding may further our understanding of multisensory integration and speaks to a universal treatment for pain and tinnitus—by selectively disrupting parahippocampal-somatosensory and parahippocampal-auditory theta–gamma activity and connectivity.

## Teaser

De Ridder *et al*. report that pain and tinnitus share common abnormal activity and connectivity in key brain areas involved in sensory and memory processing. The difference between auditory and somatosensory phantom percepts results from a vicious loop of memory-auditory versus memory-sensory belief updating in the context of missing input.

## Graphical Abstract

**Graphical abstract:**
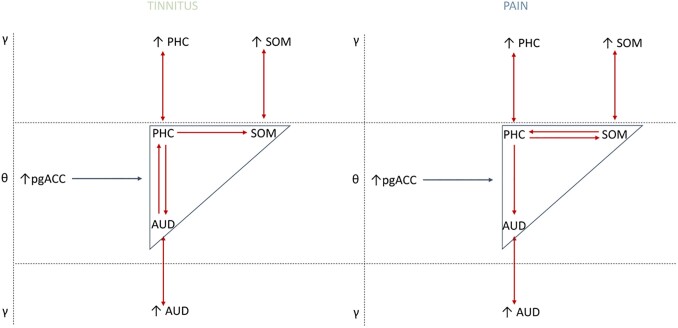

## Introduction

Multiple pathophysiological models have been proposed for tinnitus and pain, identifying common and overlapping mechanisms.^1-11^ This reflects the similar clinical characteristics of pain and tinnitus,^1,5-7,9^ and has fostered the development of similar treatment approaches.^12^ For example, thalamocortical dysrhythmia, i.e. theta–gamma cross-frequency coupling has been shown to be of relevance for both pain and tinnitus, as well as their common co-morbidity in depression.^4,10,11^ The amount of auditory gamma-band activity correlates with the loudness of tinnitus and is characteristically nested in theta activity.^13,14^ Similarly, somatosensory gamma activity correlates with the intensity of pain,^15^ and is also nested in theta activity.^10^ In this work, we test the hypothesis that these electrophysiological correlates of message passing—in the auditory and somatosensory hierarchies—reflect (Bayesian) belief updating that underwrites the experience of pain and tinnitus.

There is growing evidence for ‘Bayesian’ accounts of brain function, such as predictive coding, in which perception depends upon internal models of the sensed up world or environment, which are updated and refined based on sensory input from the different modalities.^3,16-20^ Bayesian perception involves both automatic stimulus-based perceptual inference and active response-based inference that underwrites the active sampling of predicted sensory stimuli.^20,21^ This kind of inference is thought to mediate Bayesian belief updating via the exchange of (top-down) predictions and (bottom-up) precision-weighted prediction errors among neuronal populations in sensorimotor hierarchies that have distinct electrophysiological correlates. Top-down predictions have been associated with beta band activity,^22-25^ the precision of predictions with alpha activity,^25^ and prediction errors with gamma oscillatory activity in sensory cortex.^26-32^ Predictions in active inference have also been associated with theta activity,^33^ precision with delta,^34^ and prediction errors with gamma activity in hippocampus and related cortical areas.

Two pathophysiological models for tinnitus have been proposed that are based on belief representations of the environment in the setting of auditory uncertainty.^3,19,35,36^ One account postulates that tinnitus is the result of memory-based prior beliefs about sounds, which are mobilized in compensation for the absence of evidence from sensory input.^3,19,35,36^ Alternatively, spontaneous activity in the ascending auditory pathway (a ‘tinnitus precursor’) provides evidence against the default prediction of ‘no sound’, and chronic tinnitus occurs when this activity gains sufficient ‘precision’ to influence perceptual inference or updating higher in the hierarchy.^24,37^ Both accounts hypothesise an increase in precision-weighted prediction errors at various levels of the auditory hierarchy that, neurophysiologically, would correspond to an increase in the postsynaptic gain or excitability of neuronal populations broadcasting prediction errors (generally thought to be superficial pyramidal cells)^38,39^

In this predictive coding formulation, gamma-band activity in the auditory cortex has been linked to prediction error processing.^24,36,40,41^ Yet, this prediction error needs to be propagated to higher or deeper levels of hierarchical processing, before Bayesian belief updating can be phenomenologically linked to tinnitus.^42,43^ Indeed, auditory cortex activity, if not connected to default mode and frontoparietal (central executive) network activity does not reach awareness, as identified by studies involving neurochemical and traumatic disorders of consciousness.^44-47^

However, the auditory system does not work in isolation: in hierarchical inference the brain uses a kind of abductive reasoning, by means of multisensory integration, to verify whether auditory information is a true reflection of states of affairs in the outside world.^48-50^ Multisensory integration allows for more precise (i.e. confident) representations than possible with a single sensory system, by accumulating coincident and consistent evidence from each sense,^51,52^ in keeping with abductive reasoning: anecdotally, reflecting the duck test: ‘When I see a bird that walks like a duck and swims like a duck and quacks like a duck, I call that bird a duck’.^53^ This is entirely in keeping with a Bayesian account of perception, as the posterior probability of a common cause increases with congruent multisensory evidence. Multisensory integration requires multisensory interactions, which means that prediction errors detected in one modality need to be weighted in relation to the precision of sensory input in other senses. This precision engineered multisensory integration is seen between the auditory and the visual system, evident in for example lipreading, the McGurk effect and ventriloquism.^54,55^ The McGurk illusion occurs when the auditory component of one sound is paired with the visual component of another sound, leading to the perception of a third sound.^56,57^ This necessarily requires the right balance of precision for each sensory modality—and the relative precision of sensory and prior prediction errors. For example, I will attend to auditory cues in a dark room (with imprecise visual cues) but may rely upon my prior beliefs in the absence of audiovisual sensory information—leading to illusory phenomena or hallucinosis.

For example, somatosensory-auditory interactions can generate the skin parchment illusion,^58^ in which subjects rub their hands together while the sound of rubbing is recorded. When played back, the subjects report the skin on their hands turning dry as parchment.^58^ The somatosensory-auditory interaction may also be involved in suppressing self-generated sounds, e.g. in chewing,^59^ and in permitting normal speech.^60,61^ In terms of precision weighting, this would correspond to the attenuation of sensory precision, i.e. suspending attention to the consequences of movement to allow the movement to occur.^62-65^

In a recent study, the effective connectivity in tinnitus was shown to be reduced between auditory cortex and parahippocampus as well as the inferior frontal gyrus,^66^ which may reflect auditory deprivation. But increased effective connectivity was identified in tinnitus patients between frontal and auditory cortex, in comparison to controls,^66^ which may reflect an increased sensitivity to bottom-up auditory prediction errors (the effective connectivity between the parahippocampus and auditory cortex was not reported). It has been shown that—in auditory deafferentation—the brain calls on prior beliefs about the most likely cause of impoverished auditory input, e.g. priors in parahippocampal auditory memory.^41,67,68^ As such, the missing input is ‘filled in’ to optimally balance prior beliefs against the imprecise sensory evidence, associated with auditory deprivation.^19^ Assuming the parahippocampus is relevant for contextual memory processing, it can be hypothesized that multisensory prior (i.e. contextual) information is stored in the parahippocampus.^69-71^

In summary, based upon a predictive coding formulation of multisensory information, we hypothesised an abnormality of precision-weighted prediction error message-passing in two conditions that share electrophysiological and phenomenological characteristics; namely, tinnitus and chronic pain conditions. Our question was whether aberrant precision was unique to each condition—i.e. expressed at lower, modality specific, hierarchical levels—or common to both conditions—i.e. expressed that higher, modality general, hierarchical levels; such as the parahippocampal formation, or both.

Equipped with an association between characteristic electrophysiological frequencies of top-down predictions, bottom-up prediction errors, and their precision, we performed whole-brain analyses of activity, i.e. current density, followed by more local region of interest (ROI) analyses of connectivity, both functional and effective connectivity, extracted from resting-state EEGs of patients with chronic tinnitus or pain. We report the commonalities and differences between the two pathologies, in comparison to healthy controls without tinnitus or pain. We present a comprehensive analysis of both regional electrophysiological responses and coupling between ROI in source space averaged over the entire recording time. In brief, we report whole-brain analyses using statistical parametric mapping and a set of complementary measures of neuronal message passing, based upon (frequency specific) functional and effective connectivity. To protect against false positives, we used a step-down approach: (i) basing our ROI selection on whole-brain analyses for subsequent connectivity analyses, and (ii) using multivariate [multivariate analysis of covariance (MANOVA)] tests that, if significant, licensed posthoc univariate [analysis of covariance (ANOVA)] tests. Our hope was to further elucidate the mechanisms of multisensory integration in the brain that, more specifically, may speak to the development of specific treatments for tinnitus and pain.

## Methods

### Subjects

A total of 150 participants (age: 53.23 ± 11.02 years; males: 84; females: 66; all Caucasian) were recruited for this study. The healthy control group (*N* = 50; age: 54.24 ± 10.21 years; males: 29; females: 21) reported no history of neurological or neuropsychiatric disorders. Tinnitus subjects (*N* = 50; age: 51.24 ± 12.932years; males: 24; females: 26) were screened by a tinnitus specialist. Using a standardized history taking pulsatile tinnitus, Meniere’s disease, otosclerosis, and chronic headache were excluded. Meniere’s disease was broadly defined as tinnitus with associated paroxysmal vertigo and/or low frequency hearing loss (=probable Meniere). Neurological disorders such as brain tumours were also excluded. All tinnitus patients had tinnitus for more than one year. A pain specialist screened pain patients (*N* = 50; age: 53.76 ± 12.22 years; males: 31; females: 19) for neuropathic pain related to deafferentation (i.e. peripheral nerve, root, or central tract lesions), and ensured that the pain was present for more than one year. Anxiety and depression, as co-morbidities of tinnitus and pain were not excluded. The study was in accordance with the ethical standards of the Helsinki declaration (1964) and was approved by the institutional ethics committee. Data is available on reasonable request.

### EEG collection and processing

#### Data collection

EEG data were collected using a conventional procedure: recordings were made with each subject sitting upright in a modest but supportive chair in a well-lit room. The recording was about five minutes long. Using Mitsar-201 amplifiers (NovaTech, http://www.novatecheeg.com/), the EEG was captured with 19 electrodes arranged in accordance with the recommended 10–20 International arrangement. It was verified that the impedances were less than 5 kΩ. Closed eyes were used for data collection (band passed between 0.15 and 200 Hz; sampling rate: 500 Hz). To prevent alcohol- or caffeine-induced changes in the EEG 72–74, participants were instructed not to consume alcohol 24 hours before the EEG recording or caffeinated beverages one hour before the recording.^72-74^ It was requested of the participants not to alter their drug regimen.

In order to minimize potential amplification of the theta power due to drowsiness, the participants’ attentiveness was continuously measured by observing both slowing of the alpha rhythm and the presence of spindles in the EEG stream.^75^ Offline data were down-sampled to 128 Hz, bandpass filtered in the frequency range of 2–44 Hz, and then transferred into Eureka! Software,^76^ plotted, and visually reviewed for manual artifact-rejection. The EEG time series were cleaned of any episodic artifacts, including eye blinks, eye movements, clenching of the teeth, body movements, and ECG artifact. For the frequency bands delta (2–3.5 Hz), theta (4–7.5 Hz), alpha (8–12 Hz), beta (13–30 Hz), and gamma (30.5–44 Hz), average Fourier cross-spectral matrices were calculated.

#### Source localization

The intracerebral sources were reconstructed using standardized low-resolution brain electromagnetic tomography (sLORETA). Prior to using the sLORETA technique, a common average reference transformation^77^ was carried out as routine procedure. sLORETA does not make any assumptions about the number of active sources; instead, it models electric neural activity as current density (A/m2). The lead-field matrix and solution space utilized in this investigation were created using the LORETA-Key program, which is accessible for free at http://www.uzh.ch/keyinst/loreta.htm. This software applies the lead field created, which applies the boundary element approach to the MNI-152, and revisited realistic electrode coordinates (Montreal Neurological Institute, Canada). Based on probabilities provided by the Demon Atlas, the sLORETA-key anatomical template divides and labels the neocortical (including hippocampus and anterior cingulate cortex) MNI-152 space in 6239 voxels of dimension 5 mm^3^. The co-registration uses the ideal conversion between the Talairach and Tournoux space and the MNI-152 space.

#### Region of interest analysis

For our ROIs, the log-transformed electric current densities were estimated in the theta (4–7.5 Hz) and gamma frequency bands (30.5–44 Hz). The left and right parahippocampus, the left and right auditory cortex, the left and right somatosensory cortex, and the pregenual anterior cingulate cortex were the ROIs in the current investigation. These ROIs were chosen based on the variations in activity that the whole-brain study identified. At each time step, the power in all 6239 voxels was normalized to a power of 1, then log transformed. Thus, for each frequency, the numbers for the ROI represent the log-transformed fraction of the overall power across all voxels. We just utilized one voxel because each ROI has a voxel size of 5 mm^3^. We do not distinguish between left and right in the pregenual anterior cingulate cortex because of how close they are to the midline. Volume conduction makes it more difficult to distinguish laterality in regions near the midline. Under the limits of whole-brain analysis, these ROIs and frequency bands were chosen based on our hypothesis, which was presented in the introduction (a priori) (please see below).

#### Lagged phase coherence

Typically, coherence and phase synchronization are viewed as signs of ‘connectivity’ between time series pertaining to various ROIs. However, a quick, non-physiological input from volume conduction heavily contaminates any measure of dependency.^78^ The confounding effect of volume conduction was completely eliminated by Pascual-Marqui's^79^ introduction of measurements of coherence and phase synchronization that maintain only non-instantaneous (delayed) connection. The degree of cross-talk between the regions generating the source activity can be inferred from this ‘delayed phase coherence’ between two sources.^10,80,81^ Cross-talk can be understood as information sharing by axonal transmission, or neural message passing, because the two components oscillate coherently with a phase lag. Specifically, the discrete Fourier transform breaks the signal down into a finite number of cosine and sine waves at the Fourier frequencies. The cosine waves lag behind their sine counterparts by a quarter of the period, which is inversely proportional to frequency; for instance, the period of a sinusoidal wave at 10 Hz is 100 ms. The sine is moved by 25 ms, or one-fourth of a cycle, in relation to the cosine. Lagged phase coherence thus shows coherent oscillations with a 25 ms delay at 10 Hz, 12.5 ms at 20 Hz, etc. According to asymptotic calculations, the threshold of significance for a particular lagged phase coherence value can be found. Measures of the multivariate time series’ linear coherence (dependency) were also assessed. When there is independence, these non-negative measurements take the value zero. The next terms—delta (2–3.5 Hz), theta (4–7.5 Hz), alpha (8–12 Hz), beta (13–30 Hz), and gamma (30.5–44 Hz)—were defined in the frequency domain.

#### Granger causality

By measuring how well the signal in the seed region can predict the signal in the target region, Granger causality measures the level of effective connectedness (i.e. directed interactions) between two regions.^82,83^ Or put another way, it qualifies as a directed functional connection. Granger causality is based on the formulation of a multivariate autoregressive model, which is then used to calculate the appropriate partial coherences after all irrelevant connections have been set to zero. In order to identify the coupling among empirically sampled neural systems, we choose to apply Granger causality, which can be directly applied to any time series.^84^

The advantages of Granger causality in furnishing frequency-dependent and multivariate measures have been clearly demonstrated in previous electrophysiology research.^85,86^ All technical details can be found in Stokes and Purdon.^87^ In general, the autoregressive coefficients correspond to Granger causality.^88,89^ The significance of Granger causality is defined as the log-ratio between the error variance of a reduced model, which predicts one time series based only on its own past values, and that of the full model, which in addition includes the past values of another time series. Remember that Granger causality does not indicate anatomical connectivity between regions but rather functional coupling between two sources that may be mediated by polysynaptic connections (i.e. vicariously via intermediate sources or ROIs). In this study, we focused on the functional connectivity for the theta frequency band between the left and right parahippocampus, left and right auditory cortex, left and right somatosensory cortex, and posterior cingulate cortex. Based on the analysis of functional connectivity measurements presented above, we chose the theta frequency band.

#### Cross-frequency coupling

A useful indicator of non-linear coupling between cortically distant areas is theta–gamma coupling (e.g. through nesting).^90^ Phase-amplitude cross-frequency coupling was used to assess this theta–gamma nesting in the left and right parahippocampus, left and right auditory cortex, and left and right somatosensory cortex. Phase-amplitude cross-frequency coupling was selected in favour of power-power because the former has been demonstrated to mirror a physiological mechanism for electrophysiological coupling in the human brain.^90^ The time-series for the *x*, *y*, and *z* components of the sLORETA current for each voxel of each ROI were first obtained in order to compute nesting. These are the three orthogonal directions in space's three electrical current time series. Then, these were band-pass filtered in the theta (4–7.5 Hz) and gamma (30.5–44 Hz) frequency ranges. A principal component analysis was performed for the overall *x*, *y*, and *z* component in each frequency band and for each ROI. For the theta and beta/gamma bands, the first component was kept. The signal envelope was kept while the gamma component's Hilbert transform was computed. Keep in mind that each source or ROI has nested dynamics that are indexed by cross frequency coupling (as opposed to connectivity between sources).

### Statistical analysis

#### Whole brain

The methodology used was statistical parametric mapping. This is based on estimating, via randomization, the empirical probability distribution for the max-statistic under the null hypothesis.^91^ This methodology corrects for multiple testing (i.e. for the collection of tests performed for all voxels, and for all frequency bands). Due to the non-parametric nature of this method, its validity does not rely on any assumption of Gaussianity.^91^ These whole-brain comparisons were performed by sLORETA through multiple voxel-by-voxel comparisons using a logarithm of *F*-ratio. The significance threshold for all tests was based on a permutation test with 5000 permutations. Comparisons were made between the tinnitus and non-tinnitus subject groups.

#### Whole-brain conjunction analysis

We performed a combination analysis in addition to the group comparison of the tinnitus and pain individuals.^92-95^ By locating regions that are active during independent subtraction, a conjunction analysis can determine a ‘shared processing component’ for two or more tasks/situations.^92-95^ Although general conjunction analysis is utilized in a within group condition, Friston *et al*.^93^ also stated that it can be applied between groups, for example.^96,97^ To leave just pathological activity (activity that differed from the healthy individuals) for the tinnitus and pain subjects, we chose to subtract the control group from the tinnitus and pain subjects. We performed a conjunction analysis on the tinnitus and pain participants to determine the regional pathologies they share.

#### Region of interest

We used the pregenual anterior cingulate cortex's log-transformed current density for the theta frequency band as dependent variables and the group (controls, tinnitus, and pain) as independent variables in a MANOVA. Additionally, we conducted a MANOVA using the log-transformed current density for the gamma frequency band for the left and right parahippocampus, left and right auditory cortex, and left and right somatosensory cortex as dependent variables and group (controls, tinnitus, and pain) as independent variables. If the MANOVA result was significant, a univariate ANOVA was carried out independently for each region. To account for the various univariate ANOVAs, the Holm–Bonferroni multiple correction procedure was used.^98^

#### Lagged phase coherence

Lagged phase coherence or functional connectivity comparison between tinnitus and non-tinnitus patients were calculated for the various frequency bands (delta, theta, alpha, beta, and gamma). Based on a permutation test with 5000 permutations, the significance threshold was determined. Multiple testing is corrected using this process (i.e. for the collection of tests performed for all voxels, and for all frequency bands).

#### Whole-brain lagged phase coherence

We performed a conjunction connectivity analysis^92-95^ to further contrast tinnitus and pain individuals. In order to leave just pathological connection (connectivity that diverged from the healthy individuals) for the tinnitus and pain subjects, we chose to subtract the control group from the tinnitus and pain patients. We performed a conjunction connectivity analysis on for the tinnitus and pain participants to determine what pathological connectivity they share.

#### Granger causality

A MANOVA including the Granger causality for the effective connectivity (pgACC→left AUD, pgACC→left AUD, pgACC→left SOM, pgACC→right SOM, pgACC→left PHC, pgACC→right PHC, left AUD→pgACC, right AUD→pgACC, left SOM→pgACC, right SOM→pgACC, left PHC→pgACC, right PHC→pgACC) as dependent variables and group (controls, tinnitus and pain) as independent variables for the theta frequency band was conducted. If the outcome of the MANOVA was significant, a MANOVA was conducted for the pgACC→left AUD and left AUD→pgACC, for the pgACC→right AUD and right AUD→pgACC, for the pgACC→left SOM and left SOM→pgACC, for the pgACC→right SOM and right SOM→pgACC, for the pgACC→left PHC and left PHC→pgACC, and for the pgACC→right PHC, and right PHC→pgACC, separately. If the outcome of the MANOVA was significant, a one-way ANOVA was applied that was further explored if significant using a pairwise comparison. A correction for multiple comparisons using the Holm–Bonferroni method was applied to correct for the different univariate ANOVAs.^98^

Furthermore, a MANOVA including the Granger causality for the effective connectivity (left AUD→ left PHC, left PHC→left AUD, right AUD→ right PHC, right PHC→right AUD, left SOM→ left PHC, left PHC→left SOM, right SOM→ right PHC, right PHC→right SOM) as dependent variables and group (controls, tinnitus and pain) as independent variables for the theta frequency band was conducted. If the outcome of the MANOVA was significant, a MANOVA was conducted for the left AUD→ left PHC and left PHC→left AUD, for the right AUD→ right PHC and right PHC→right AUD, for the left SOM→ left PHC and left PHC→left SOM, and for the right SOM→ right PHC and right PHC→right SOM, separately. If the outcome of the MANOVA was significant, a one-way ANOVA was applied that was further explored if significant using a pairwise comparison. A correction for multiple comparisons using the Holm–Bonferroni method was applied to correct for the different univariate ANOVAs.^98^

Finally, a MANOVA including the effective connectivity (left PHC→right PHC, right PHC→left PHC) as dependent variables and group (controls, tinnitus and pain) as independent variables for the theta frequency band was conducted. If the outcome of the MANOVA was significant, a one-way ANOVA was applied that was further explored, if significant, using a pairwise comparison. A correction for multiple comparisons using the Holm–Bonferroni method was applied to correct for the different univariate ANOVAs.^98^

#### Phase-amplitude coupling

We performed a MANOVA including the phase-amplitude coupling for theta–gamma as dependent variables for left and right auditory cortex, left and right somatosensory cortex, and the left and right parahippocampus for gamma frequency band as dependent variables and group (controls, tinnitus and pain). If the outcome of the MANOVA was significant, a univariate ANOVA was conducted for the different cross-frequency couplings separately, using the Holm–Bonferroni correction for multiple comparisons.^98^

### Data availability

The data used in this study are not publicly available, although the authors are willing to share it upon reasonable request.

## Results

### Whole-brain analysis

#### Tinnitus versus control subjects

**Figure 1:**
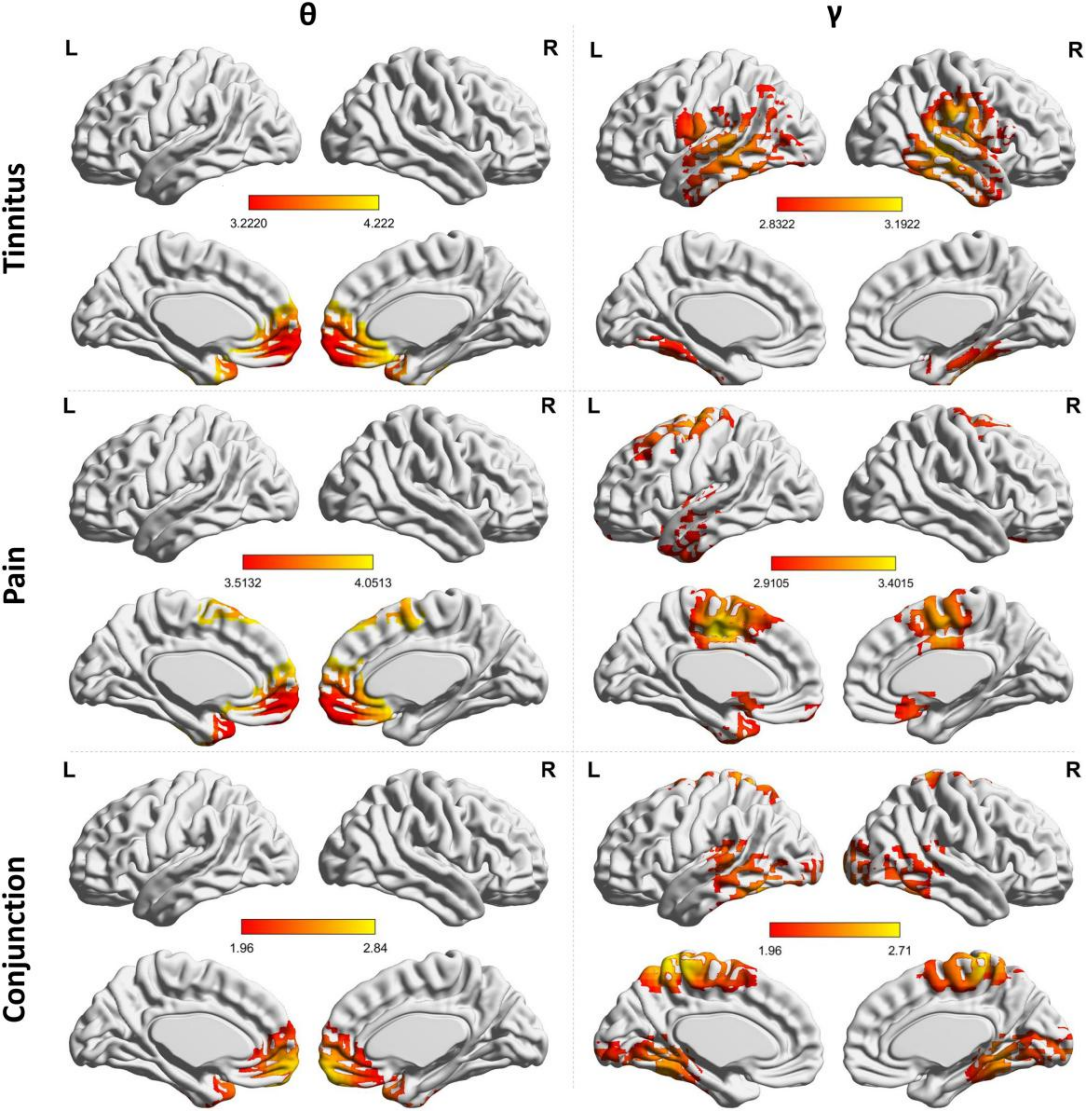
**Activity.** *Top left*: A comparison between tinnitus and control subjects demonstrates an increased activity in the pregenual anterior cingulate cortex for the theta frequency band for the tinnitus subjects (statistics: permutation testing, *F-ratio)*. *Top right*: increased gamma frequency band activity in the auditory cortex extending into the left and right somatosensory and motor cortex in tinnitus (statistics: permutation testing, *F-ratio)*. *Middle left*: Subjects with neuropathic pain demonstrates an increased activity in the pregenual anterior cingulate cortex extending into the ventral medial prefrontal cortex for the theta frequency band subjects (statistics: permutation testing, *F-ratio)*. *Middle right*: Increased activity was revealed in the left and right somatosensory and motor cortex as well as the subgenual anterior cingulate cortex for pain subjects (statistics: permutation testing, *F-ratio)*. *Bottom left and right:* A conjunction between neuropathic pain and tinnitus (after subtraction of activity in controls) yields an effect for both the theta and gamma frequency band. For the theta frequency band, increased activity in the pregenual anterior cingulate cortex extending into the ventral medial prefrontal cortex is identified, while for the gamma frequency band increased activity was also found in the left and right somatosensory cortex extending into motor cortex, the left and right auditory cortex and the left and right parahippocampus for the gamma frequency band (statistics: *Z-score)*.

A comparison between tinnitus and control subjects demonstrates significantly increased activity in the pregenual anterior cingulate cortex extending into the ventral medial prefrontal cortex for the theta frequency band for the tinnitus subjects (*F* = 3.22, *P* < 0.05). For the gamma frequency band, we found significantly increased activity in the auditory cortex extending into the left and right somatosensory and motor cortex (*F* = 2.83, *P* < 0.05). No significant effects were observed for the delta, alpha, and beta frequency bands. See Fig. 1 for an overview.

#### Pain versus Control subjects

Subjects with neuropathic pain demonstrated significantly increased activity in the pregenual anterior cingulate cortex extending into the ventral medial prefrontal cortex for the theta frequency band in comparison to control subjects (*F* = 3.51, *P* < 0.05). Furthermore, increased gamma-band activity was revealed in the left and right somatosensory and motor cortex as well as the subgenual anterior cingulate cortex for pain subjects in comparison to control subjects (*F* = 2.91, *P* < 0.05). No significant effects were observed for the delta, alpha, and beta frequency bands. See Fig. 1 for overview.

#### Conjunction between pain and tinnitus subjects

A conjunction between neuropathic pain and tinnitus (after subtraction of activity in controls) yielded a significant effect for both the theta and gamma frequency band. For the theta frequency band, we found increased activity in the pregenual anterior cingulate cortex extending into the ventral medial prefrontal cortex (*Z* = 1.96, *P* < 0.05). Increased activity was also found in the left and right somatosensory cortex extending into motor cortex, the left and right auditory cortex and the left and right parahippocampus for the gamma frequency band (*Z* = 1.96, *P* < 0.05). No significant effects were observed for the delta, alpha, and beta frequency bands. See Fig. 1 for overview.

### Region of interest analyses

To characterise the differences between chronic tinnitus and neuropathic pain, we conducted a ROI analysis for the theta frequency band, including the pregenual anterior cingulate cortex and—for the gamma frequency band—the left and right auditory cortex, the left and right somatosensory cortex, and left and right parahippocampus. The selection of these frequency bands and ROIs was based on our hypothesis and confirmed (*post hoc*) by the whole-brain analysis.

**Figure 2:**
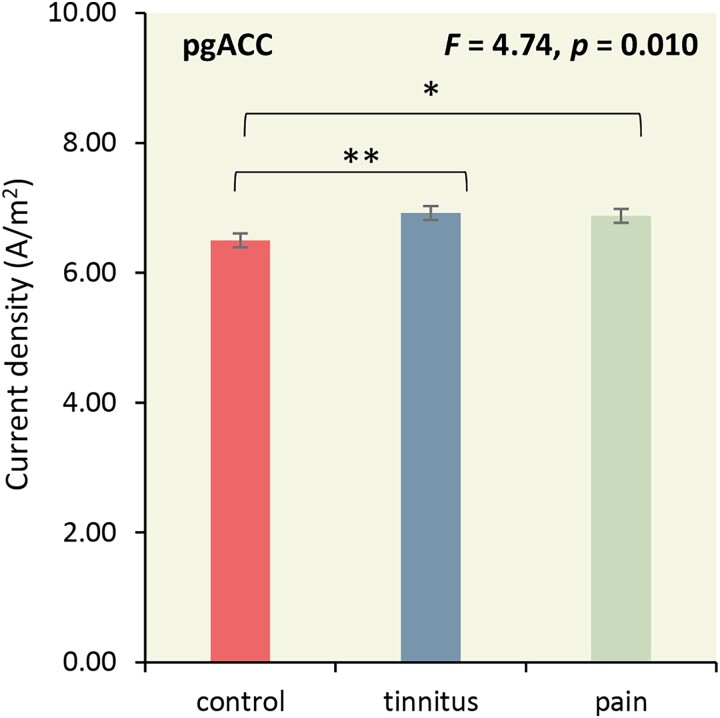
**The role of the pregenual anterior cingulate cortex.** A pairwise comparison revealed an increased current density for tinnitus subjects and pain subjects in comparison to controls for the pregenual anterior cingulate cortex (pgACC) subjects (statistics: ANOVA*)*. *P < 0.05; **P < 0.01.

An ANOVA of the log-transformed current density for the pregenual anterior cingulate cortex as dependent variable and group (controls, tinnitus and pain) as independent variables for the theta frequency band showed an overall effect (*F* = 4.74, *P* = 0.010, *η^2^* = 0.06; see Fig. 2). A pairwise comparison revealed an increased current density for tinnitus subjects (*F* = 7.84, *P* = 0.006, *η^2^* = 0.05) and pain subjects (*F* = 6.30, *P* = 0.013, *η^2^* = 0.04) in comparison to controls. No significant difference was obtained between tinnitus and pain subjects (*F* = 0.08, *P* = 0.77, *η^2^* = 0.001). These effects remained after Holms–Bonferroni correction.

**Figure 3:**
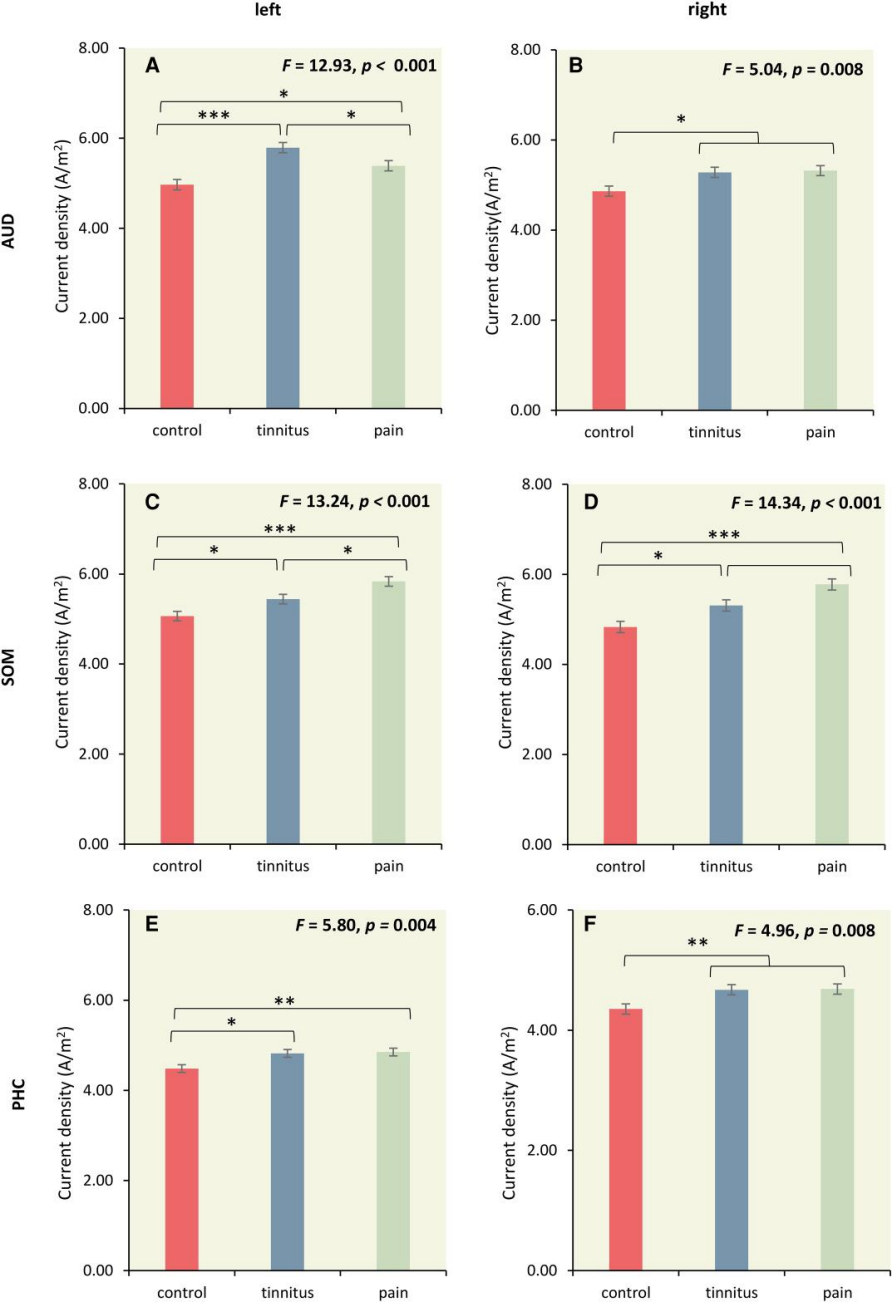
**The role of the auditory, somatosensory, and parahippocampal cortex.** (**A-B**) A pairwise comparison revealed an increased current density for both tinnitus subjects and pain subjects in comparison to controls at the auditory cortex. Furthermore, tinnitus subjects had increased current density for the left auditory cortex in comparison to pain subjects. No significant difference was revealed between tinnitus and pain subjects for the right auditory cortex. (statistics: univariate ANOVA) (**C-D**) For both the left and right somatosensory cortex (SOM) increased current density is identified in pain subjects and tinnitus subjects in comparison to controls. Pain subjects also had increased current density in comparison to tinnitus subjects (statistics: univariate ANOVA). (**E**) The left parahippocampus (PHC) revealed an increased current density for tinnitus subjects and pain subjects in comparison to controls. (**F**) The right parahippocampus revealed an increased current density for both tinnitus subjects and pain subjects in comparison to controls (statistics: univariate ANOVA). *P < 0.05; **P < 0.01, ***P < 0.001.

A MANOVA of the log-transformed current density for the left and right auditory cortex, the left and right somatosensory cortex, and left and right parahippocampus as dependent variables and group (controls, tinnitus, and pain) as independent variables for the theta frequency band showed an overall effect (*F* = 6.15, *P* < 0.001, *η^2^* = 0.21; see Fig. 3). An univariate ANOVA revealed a significant effect left and right auditory cortex (left: *F* = 12.93, *P* < 0.001, *η^2^* = 0.15│right: *F* = 5.04, *P* = 0.008, *η^2^* = 0.06; see Figs 3A and B), the left and right somatosensory cortex (left: *F* = 13.24, *P* < 0.001, *η^2^* = 0.15│right: *F* = 14.34, *P* = 0.001, *η^2^* = 0.16; see Figs 3C and D) and left and right parahippocampus (left: *F* = 5.80, *P* = 0.004, *η^2^* = 0.07│right: *F* = 4.96, *P* = 0.008, *η^2^* = 0.06; see Figs 3D and E).

For the left auditory cortex, a pairwise comparison revealed an increased current density for tinnitus subjects (*F* = 25.85, *P* < 0.001, *η^2^* = 0.15) and pain subjects (*F* = 6.76, *P* = 0.010, *η^2^* = 0.04) in comparison to controls. Tinnitus subjects had increased current density for the left auditory cortex in comparison to pain subjects (*F* = 6.18, *P* = 0.014, *η^2^* = 0.04). For the right auditory cortex, a pairwise comparison yielded a significantly increased current density for tinnitus subjects (*F* = 6.86, *P* = 0.010, *η^2^* = 0.05) and pain subjects (*F* = 8.21, *P* = 0.005, *η^2^* = 0.05) in comparison to controls. No significant difference was revealed between tinnitus and pain subjects for the right auditory cortex (*F* = 0.06, *P* = 0.81, *η^2^* = 0.001).

For both the left and right somatosensory cortex, a pairwise comparison revealed an increased current density for pain subjects (left: *F* = 26.45, *P* < 0.001, *η^2^* = 0.15│right: *F* = 28.68, *P* < 0.001, *η^2^* = 0.16) and tinnitus subjects (left: *F* = 6.37, *P* < 0.013, *η^2^* = 0.04│right: *F* = 7.35, *P* = 0.008, *η^2^* = 0.05) in comparison to controls. Pain subjects had increased current density for the left somatosensory cortex in comparison to tinnitus subjects (left: *F* = 6.90, *P* = 0.010, *η^2^* = 0.05│right: *F* = 6.99, *P* = 0.009, *η^2^* = 0.05).

For the left parahippocampus, a pairwise comparison revealed an increased current density for tinnitus subjects (*F* = 8.01, *P* = 0.005, *η^2^* = 0.05) and pain subjects (*F* = 9.33, *P* = 0.003, *η^2^* = 0.06) in comparison to controls. No significant difference was obtained between tinnitus and pain subjects (*F* = 0.05, *P* = 0.82, *η^2^* = 0.001). For the right parahippocampus, a pairwise comparison revealed an increased current density for both tinnitus subjects (*F* = 7.15, *P* = 0.008, *η^2^* = 0.05) and pain subjects (*F* = 7.71, *P* = 0.006, *η^2^* = 0.05) in comparison to controls. No significant difference was obtained between tinnitus and pain subjects (*F* = 0.01, *P* = 0.92, *η^2^* = 0.001).

### Functional connectivity

#### Tinnitus versus Control subjects

**Figure 4:**
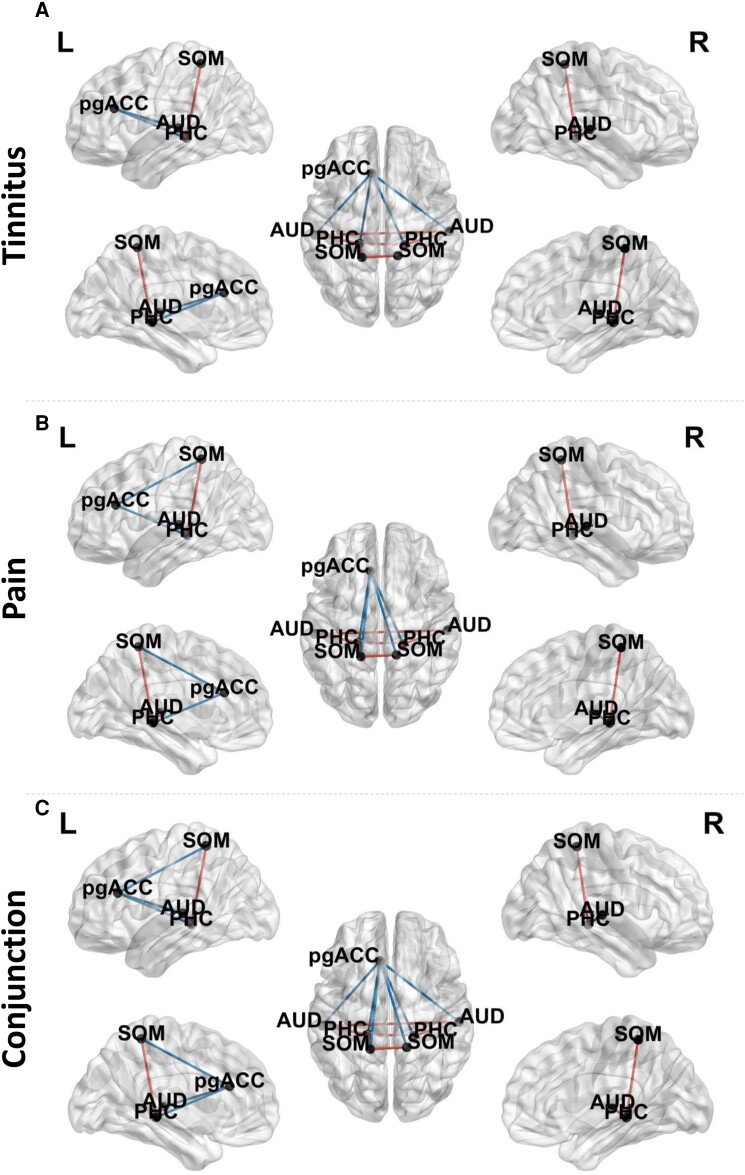
**Connectivity.** (**A**). A comparison between tinnitus and control subjects revealed significantly decreased connectivity between the pregenual anterior cingulate cortex (pgACC), and the left and right auditory cortex (AUD) and left and right somatosensory cortex (SOM), respectively. Increased connectivity was further revealed between the left and right auditory cortex as well as between the left and right somatosensory cortex and between the left and right parahippocampus for tinnitus subjects in comparison to control subjects. Furthermore, increased connectivity was seen between the left auditory cortex and the left somatosensory cortex as well as the left auditory cortex and the left parahippocampus (PHC), and the left somatosensory cortex and the left parahippocampus for the theta frequency band for the tinnitus subjects. Similar differences in connectivity were revealed for the right hemisphere between the auditory cortex, somatosensory cortex and parahippocampus for the theta frequency band (statistics: permutation testing, *F-ratio)*. (**B**). A comparison between pain and control subjects demonstrated significantly decreased connectivity between the pregenual anterior cingulate cortex, and the left and right somatosensory cortex and left and right auditory cortex, respectively. Increased connectivity was further revealed between the left and right somatosensory cortex as well as between the left and right auditory cortex and between the left and right parahippocampus for pain subjects in comparison to control subjects. Furthermore, increased connectivity was seen between the left auditory cortex and the left somatosensory cortex as well as the left auditory cortex and the left parahippocampus, and the left somatosensory cortex and the left parahippocampus for the theta frequency band for the pain subjects. Similar connections were revealed for the right hemisphere between the auditory cortex, somatosensory cortex and parahippocampus for the theta frequency band (statistics: permutation testing, *F-ratio)*. (**C**). A conjunction between neuropathic pain and tinnitus after subtraction of controls yielded a significant effect for the theta frequency band. Decreased connectivity was identified between the pregenual anterior cingulate cortex, and the left and right somatosensory cortex and left and right auditory cortex, respectively. Increased connectivity was further revealed between the left and right somatosensory cortex as well as between the left and right auditory cortex and between the left and right parahippocampus for pain and tinnitus subjects in comparison to control subjects. Furthermore, increased connectivity was found between the left auditory cortex and the left somatosensory cortex, as well as the left auditory cortex and the left parahippocampus, and the left somatosensory cortex and the left parahippocampus for the theta frequency band for the pain and tinnitus subjects. Similar differences in connectivity were found for the right hemisphere between the auditory cortex, somatosensory cortex and parahippocampus for the theta frequency band (statistics: permutation testing, *F-ratio)*.

A comparison between tinnitus and control subjects revealed significantly decreased connectivity between the pregenual anterior cingulate cortex, and the left and right auditory cortex and left and right somatosensory cortex, respectively (*F* = 3.54, *P* < 0.05). Increased connectivity was further revealed between the left and right auditory cortex as well as between the left and right somatosensory cortex and between the left and right parahippocampus for tinnitus subjects in comparison to control subjects. Furthermore, increased connectivity was seen between the left auditory cortex and the left somatosensory cortex as well as the left auditory cortex and the left parahippocampus, and the left somatosensory cortex and the left parahippocampus for the theta frequency band for the tinnitus subjects. Similar differences in connectivity were revealed for the right hemisphere between the auditory cortex, somatosensory cortex and parahippocampus for the theta frequency band. No significant effects were observed for the delta, alpha, beta, and gamma frequency bands. See Fig. 4 for overview.

#### Pain versus control subjects

A comparison between pain and control subjects demonstrated significantly decreased connectivity between the pregenual anterior cingulate cortex, and the left and right somatosensory cortex and left and right auditory cortex, respectively (*F* = 4.02, *P* < 0.05). Increased connectivity was further revealed between the left and right somatosensory cortex as well as between the left and right auditory cortex and between the left and right parahippocampus for pain subjects in comparison to control subjects. Furthermore, increased connectivity was seen between the left auditory cortex and the left somatosensory cortex as well as the left auditory cortex and the left parahippocampus, and the left somatosensory cortex and the left parahippocampus for the theta frequency band for the pain subjects. Similar connections were revealed for the right hemisphere between the auditory cortex, somatosensory cortex and parahippocampus for the theta frequency band. No significant effects were observed for the delta, alpha, beta, and gamma frequency bands. See Fig. 4 for overview.

#### Conjunction between pain and tinnitus subjects

A conjunction between neuropathic pain and tinnitus after subtraction of controls yielded a significant effect for the theta frequency band (Z = 12.01, *P* < 0.05). That is, significantly decreased connectivity between the pregenual anterior cingulate cortex, and the left and right somatosensory cortex and left and right auditory cortex, respectively. Increased connectivity was further revealed between the left and right somatosensory cortex as well as between the left and right auditory cortex and between the left and right parahippocampus for pain and tinnitus subjects in comparison to control subjects. Furthermore, increased connectivity was found between the left auditory cortex and the left somatosensory cortex, as well as the left auditory cortex and the left parahippocampus, and the left somatosensory cortex and the left parahippocampus for the theta frequency band for the pain and tinnitus subjects. Similar differences in connectivity were found for the right hemisphere between the auditory cortex, somatosensory cortex and parahippocampus for the theta frequency band. No significant effects were observed for the delta, alpha, beta, and gamma frequency bands. See Fig. 4 for overview.

### Effective connectivity: Granger causality

Based on functional connectivity, we looked specifically at the directionality between the pregenual anterior cingulate cortex, and the left and right auditory cortex, the left and right somatosensory cortex, and the left right parahippocampus, respectively, between tinnitus, pain and control subjects for the theta frequency band.

#### The role of the pregenual anterior cingulate cortex

**Figure 5:**
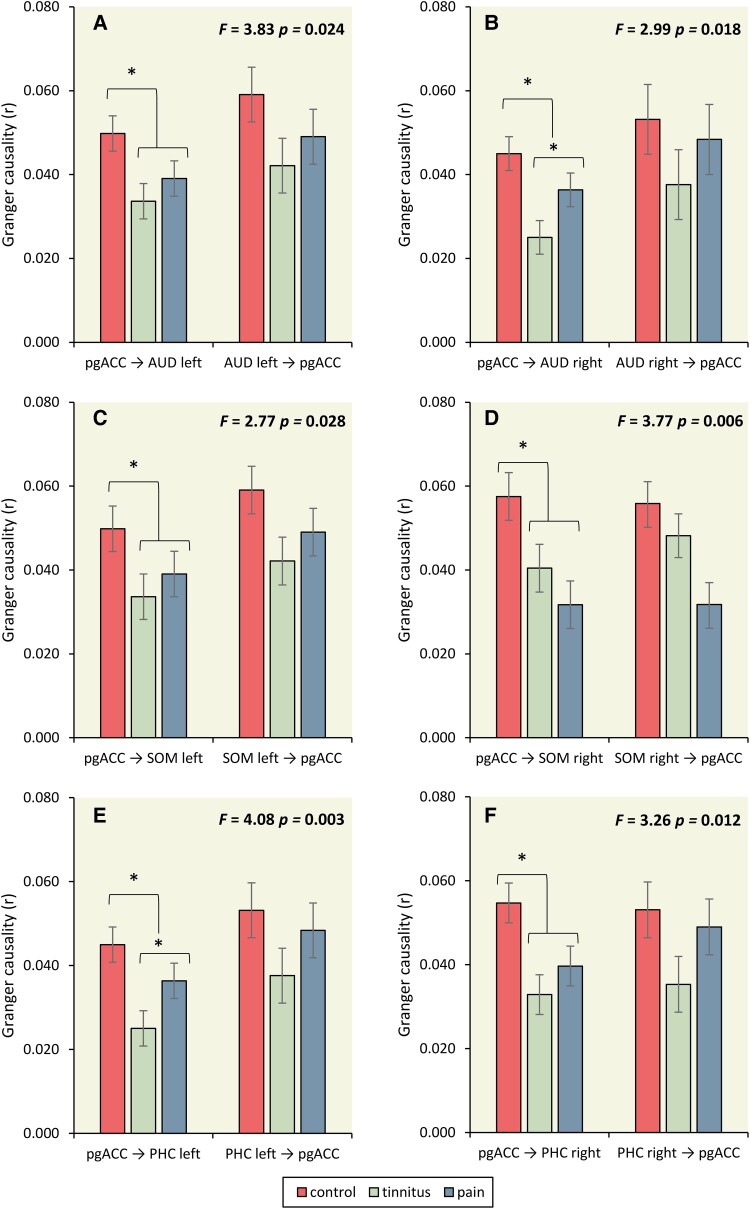
**Effective connectivity, the role of the pregenual anterior cingulate cortex**. (**A**) A significant effect for pgACC→left AUD, indicating tinnitus and pain subjects showed decreased coupling for pgACC→left AUD in comparison to controls. No difference was obtained between tinnitus and pain. A one-way ANOVA for left AUD→pgACC did not revealed a group effect (statistics: univariate ANOVA). (**B**) For the coupling between pgACC→right AUD tinnitus subjects had decreased Granger causality in comparison to control and pain subjects. Tinnitus subjects had decreased Granger causality for pgACC→right PHC in comparison to controls subjects (statistics: univariate ANOVA) (**C**) For the communication pgACC→left SOM and left SOM→pgACC, an effect was found for both the communication pgACC→left SOM and left SOM→pgACC. For pgACC→left SOM, pain subjects demonstrated significantly decreased communication in comparison to controls, but not with tinnitus patients. Tinnitus subjects did not differ from controls either. (statistics: univariate ANOVA) (**D**) For pgACC→right SOM, a decrease in communication was revealed for pain and tinnitus subjects in comparison to control subjects. No effect was found between pain and tinnitus subjects. For right SOM→pgACC, a decreased coupling was revealed for pain subjects in comparison to tinnitus and control subjects. No difference was obtained between tinnitus and control subjects. (statistics: univariate ANOVA) (**E**) A comparison revealed that both tinnitus and pain subjects have decreased communication for pgACC→left PHC in comparison to control subjects. In addition, tinnitus subjects have reduced for pgACC→left PHC in comparison to pain subjects. (**F**) A comparison revealed that both tinnitus and pain subjects have decrease in communication for pgACC→right PHC in comparison to control subjects. In addition, tinnitus subjects have reduced communication for pgACC→right PHC in comparison to pain subjects. (statistics: univariate ANOVA) *P < 0.05; pregenual anterior cingulate cortex (pgACC); auditory cortex (AUD); somatosensory cortex (SOM); parahippocampus (PHC).

A MANOVA of the Granger causality for the effective connectivity (pgACC→left AUD, pgACC→left AUD, pgACC→left SOM, pgACC→right SOM, pgACC→left PHC, pgACC→right PHC, left AUD→pgACC, right AUD→pgACC, left SOM→pgACC, right SOM→pgACC, left PHC→pgACC, right PHC→pgACC) as dependent variables and group (controls, tinnitus and pain) as independent variables for the theta frequency band showed an overall effect (*F* = 1.91, *P* = 0.008, *η^2^* = 0.14) (see Fig. 5 and Table 1).

**Table 1:** Granger causality

The role of the pregenual anterior cingulate cortex						
	Control	Tinnitus	Pain	*F*-value	*P*-value	*η* ^2^
*F* = 3.83, *P* = 0.024, *η^2^* = 0.05						
pgACC→left AUD	0.049^a^	0.033^b^	0.039^b^	3.83	0.024	0.05
left AUD→pgACC	0.059	0.042	0.049	1.70	0.186	0.23
*F* = 2.99, *P* = 0.018, *η^2^* = 0.039						
pgACC→right AUD	0.045^a^	0.025^b^	0.036^c^	3.95	0.049	0.026
right AUD→pgACC	0.053	0.037	0.048	0.83	0.362	0.006
*F* = 2.77, *P* = 0.028, *η^2^* = 0.036						
pgACC→left SOM	0.056^a^	0.047^a,b^	0.035^b^	3.85	0.023	0.050
left SOM→pgACC	0.056^a^	0.039^b^	0.034^b^	4.14	0.018	0.053
*F* = 3.77, *P* = 0.006, *η^2^* = 0.048						
pgACC→right SOM	0.057^a^	0.040^b^	0.031^b^	5.32	0.006	0.067
right SOM→pgACC	0.056^a^	0.48^a^	0.031^b^	5.56	0.005	0.070
*F* = 4.08, *P* = 0.003, *η^2^* = 0.053						
pgACC→left PHC	0.066^a^	0.039^b^	0.043^c^	7.16	0.001	0.089
left PHC→pgACC	0.058	0.039	0.049	2.47	0.086	0.032
*F* = 3.26, *P* = 0.012, *η^2^* = 0.042						
pgACC→right PHC	0.054^a^	0.032^b^	0.039^b^	5.54	0.005	0.070
right PHC→pgACC	0.053	0.035	0.049	1.96	0.144	0.026

The communication between the auditory cortex, somatosensory cortex and the parahippocampus						
	Control	Tinnitus	Pain	*F*-value	*P*-value	*η* ^2^
*F* = 4.44, *P* = 0.002, *η^2^* = 0.06						
left AUD→left PHC	0.033^a^	0.043^b^	0.031^a^	5.15	0.007	0.07
left PHC→left AUD	0.026^a^	0.027^a^	0.035^b^	4.55	0.012	0.06
*F* = 4.35, *P* = 0.002, *η^2^* = 0.07						
right AUD→pgACC	0.028^a^	0.039^b^	0.028^a^	5.14	0.007	0.065
left PHC→left AUD	0.026^a^	0.035^b^	0.035^b^	3.61	0.029	0.047
*F* = 0.329, *P* = 0.012, *η^2^* = 0.043						
left SOM→left PHC	0.009^a^	0.011^a,b^	0.015^b^	6.03	0.003	0.076
left PHC→left SOM	0.011^a^	0.014^b^	0.017^b^	4.16	0.018	0.053
*F* = 3.15 *P* = 0.0015, *η^2^* = 0.041						
right SOM→right PHC	0.013^a^	0.011^a^	0.018^b^	3.49	0.033	0.045
right PHC→right SOM	0.014^a^	0.18^a^	0.021^a,b^	3.62	0.029	0.047

Different superscripts a and b denote a statistically significant difference of *P* < 0.05.

Based on these findings, a MANOVA including the effective connectivity (pgACC→left AUD, left AUD→pgACC) as dependent variables and group (controls, tinnitus and pain) as independent variables for the theta frequency band showed an overall effect. A one-way ANOVA showed a significant effect for pgACC→left AUD, indicating tinnitus and pain subjects showed decreased coupling for pgACC→left AUD in comparison to controls. No difference was identified between tinnitus and pain. A one-way ANOVA for left AUD→pgACC did not reveal a group effect.

A MANOVA of the Granger causality pgACC→right AUD and right AUD→pgACC for the theta frequency band revealed a group effect. A one-way ANOVA showed a group effect for the connection pgACC→right AUD, but not right AUD→pgACC. For the coupling between pgACC→right AUD, a pairwise comparison revealed that tinnitus subjects had decreased Granger causality in comparison to control and pain subjects. In addition, tinnitus subjects had decreased Granger causality for pgACC→right PHC in comparison to controls subjects.

For the communication pgACC→left SOM and left SOM→pgACC, a MANOVA yielded a significant effect. A one-way ANOVA revealed a significant effect for both the communication pgACC→left SOM and left SOM→pgACC. For pgACC→left SOM, our data revealed that pain subjects had significantly decreased communication in comparison to controls, but not with tinnitus patients. Tinnitus subjects did not differ from controls either.

A MANOVA revealed a significant effect for the communication pgACC→right SOM and right SOM→pgACC. A one-way ANOVA revealed for both pgACC→right SOM and right SOM→pgACC a significant effect. For pgACC→right SOM, a decrease in communication was revealed for pain and tinnitus subjects in comparison to control subjects. No effect was found for pain and tinnitus subjects. For right SOM→pgACC, a decreased coupling was revealed for pain subjects in comparison to tinnitus and control subjects. No difference was identified between tinnitus and control subjects.

A similar analysis revealed an overall effect in the communication for pgACC→left PHC and left PHC→pgACC. A one-way ANOVA showed a significant effect for pgACC→left PHC, but not for the left PHC SOM→pgACC. A pairwise comparison revealed that both tinnitus and pain subjects have decreased communication for pgACC→left PHC in comparison to control subjects. In addition, tinnitus subjects have reduced for pgACC→left PHC in comparison to pain subjects.

A MANOVA further demonstrated a significant effect for pgACC→left PHC and left PHC →pgACC. A one-way ANOVA yielded a significant effect for pgACC→left PHC, but not for the left PHC →pgACC. A pairwise comparison revealed that both tinnitus and pain subjects have decreased communication for pgACC→left PHC in comparison to control subjects. No significant difference was identified between tinnitus and pain subjects. A similar analysis including pgACC→right PHC and right PHC →pgACC revealed the same results.

#### The communication between the auditory cortex, somatosensory cortex, and the parahippocampus

**Figure 6:**
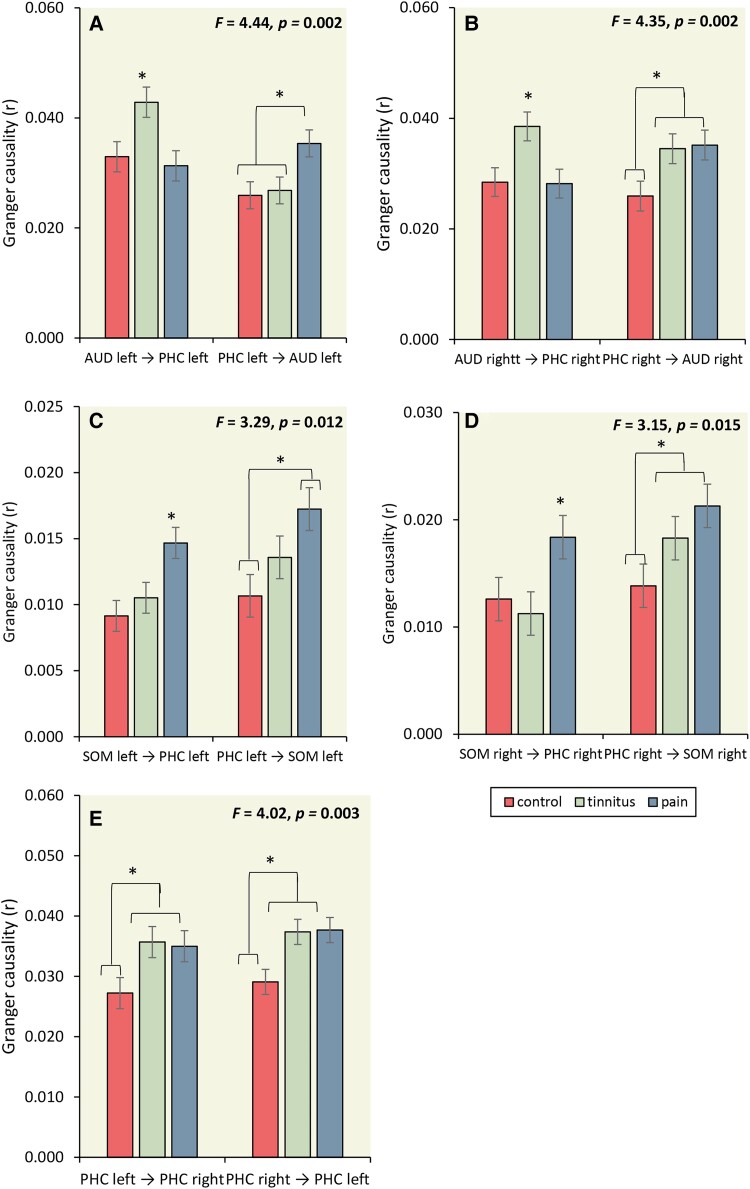
**The communication between the auditory cortex, somatosensory cortex and the parahippocampus.** (**A**) For the left AUD→ left PHC, increased significant coupling was obtained for the tinnitus subjects in comparison to pain and control subjects. No effect was obtained between pain and control subjects. For left PHC→left AUD, a significant effect showed an increase in coupling for pain subjects in comparison to tinnitus and control subjects. No difference was obtained between tinnitus and control subjects. (statistics: univariate ANOVA) (**B**) A comparison showed that for right AUD→ right PHC tinnitus subjects showed increased coupling in comparison to control and pain subjects. No difference was revealed between control and pain subjects. For the right PHC→right AUD, a comparison showed that both tinnitus and pain subjects have increased Granger causality in comparison to control and that there was no difference between tinnitus and pain subjects. (statistics: univariate ANOVA) (**C**) For the left SOM→ left PHC, increased significant coupling was found for the tinnitus subjects in comparison to control subjects. No effect was seen between pain and control subjects, or between pain and tinnitus subjects. For left PHC→left SOM, a significant effect showed increased coupling for both tinnitus and pain subjects in comparison to control subjects. No difference was obtained between tinnitus and pain subjects. (statistics: univariate ANOVA) (**D**) A pairwise comparison showed that for both right SOM→ right PHC and right PHC→right SOM that pain subjects showed increased coupling in comparison to tinnitus and control subjects. No difference was revealed between tinnitus and control subjects. (statistics: univariate ANOVA) (**E**) Coupling between the left and right parahippocampus, indicating tinnitus and pain subjects showed increased communication in comparison to controls. No difference was obtained between tinnitus and pain. (statistics: univariate ANOVA) *P < 0.05; pregenual anterior cingulate cortex (pgACC); auditory cortex (AUD); somatosensory cortex (SOM); parahippocampus (PHC).

A MANOVA of the Granger causality for the effective connectivity (left AUD→ left PHC, left PHC→left AUD, right AUD→ right PHC, right PHC→right AUD, left SOM→ left PHC, left PHC→left SOM, right SOM→ right PHC, right PHC→right SOM) as dependent variables and group (controls, tinnitus and pain) as independent variables for the theta frequency band showed an overall effect (*F* = 2.80, *P* < 0.001, *η^2^* = 0.14) (see Fig. 6 and Table 1).

A MANOVA for the coupling between left AUD→ left PHC and left PHC→left AUD revealed a significant effect. A one-way ANOVA showed both left AUD→ left PHC and left PHC→left AUD were significant. For the left AUD→ left PHC, increased significant coupling was obtained for the tinnitus subjects in comparison to pain and control subjects. No effect was identified between pain and control subjects. For left PHC→left AUD, a significant effect showed an increase in coupling for pain subjects in comparison to tinnitus and control subjects. No difference was identified between tinnitus and control subjects.

For the coupling between right AUD→ right PHC and right PHC→right AUD, an overall significant effect was revealed. Both right AUD→ right PHC and right PHC→right AUD were significant. A pairwise comparison showed that for right AUD→ right PHC tinnitus subjects showed increased coupling in comparison to control and pain subjects. No difference was revealed between control and pain subjects. For the right PHC→right AUD, a comparison showed that both tinnitus and pain subjects have increased Granger causality in comparison to controls and that there was no difference between tinnitus and pain subjects.

A significant overall effect was identified for the coupling between left SOM→ left PHC and left PHC→left SOM. A one-way ANOVA showed both left SOM→ left PHC and left PHC→left SOM were significant. For the left SOM→ left PHC, increased significant coupling was found for the tinnitus subjects in comparison control subjects. No effect was seen between pain and control subjects, or between pain and tinnitus subjects. For left PHC→left SOM, a significant effect showed increased coupling for both tinnitus and pain subjects in comparison to control subjects. No difference was identified between tinnitus and pain subjects.

For the communication for right SOM→ right PHC and right PHC→right SOM again, an overall significant effect was demonstrated. Both right SOM→ right PHC and right PHC→right SOM were significant. A pairwise comparison showed that for both right SOM→right PHC and right PHC→right SOM in pain subjects showed increased coupling in comparison to tinnitus and control subjects. No difference was revealed between tinnitus and control subjects.

#### Coupling between the left and right parahippocampus

A MANOVA of the effective connectivity (left PHC→right PHC, right PHC→left PHC) as dependent variables and group (controls, tinnitus and pain) as independent variables for the theta frequency band showed an overall effect (*F* = 4.00, *P* = 0.004, *η^2^* = 0.07). A one-way ANOVA showed a significant effect for both left PHC→right PHC (*F* = 5.15, *P* = 0.007, *η^2^* = 0.065) and right PHC→left PHC (*F* = 4.32, *P* = 0.015, *η^2^* = 0.055), indicating tinnitus (left PHC→right PHC:*F* = 8.36, *P* = 0.004, *η^2^* = 0.054│ right PHC→left PHC: *F* = 6.25, *P* = 0.014, *η^2^* = 0.041) and pain (left PHC→right PHC: *F* = 7.03, *P* = 0.009, *η^2^* = 0.046│ right PHC→left PHC: *F* = 6.70, *P* = 0.011, *η^2^* = 0.044) subjects showed increased communication in comparison to controls. No difference was identified between tinnitus and pain (left PHC→right PHC: *F* = 0.06, *P* = 0.96, *η^2^* = 0.001│ right PHC→left PHC: *F* = 0.008, *P* = 0.93, *η^2^* = 0.001). See Fig. 6.

### Theta–Gamma phase-amplitude coupling

We used theta–gamma phase-amplitude coupling to further characterise the connectivity between the left and right auditory cortex, left and right somatosensory cortex, and left and right parahippocampus for tinnitus, pain, and control subjects at the theta–gamma coupling. Our findings above showed increased activity in the gamma frequency band for left and right auditory cortex, left and right somatosensory cortex, and left and right parahippocampus for tinnitus and/or pain subjects and increased effective connectivity between the left and right auditory cortex, left and right somatosensory cortex, and left and right parahippocampus for tinnitus and pain subjects, in the theta frequency band. This suggests that interregional coupling is expressed in the theta frequency band and that local activity is generated in gamma activity. Previous research indicates that theta–gamma coupling is an effective marker of nonlinear communication between cortically distant areas,^90^ where low frequencies are proposed to function as carrier waves on top of which high frequencies are nested; presumably conveying neuronal messages that mediate belief updating.^90,99-103^

**Figure 7:**
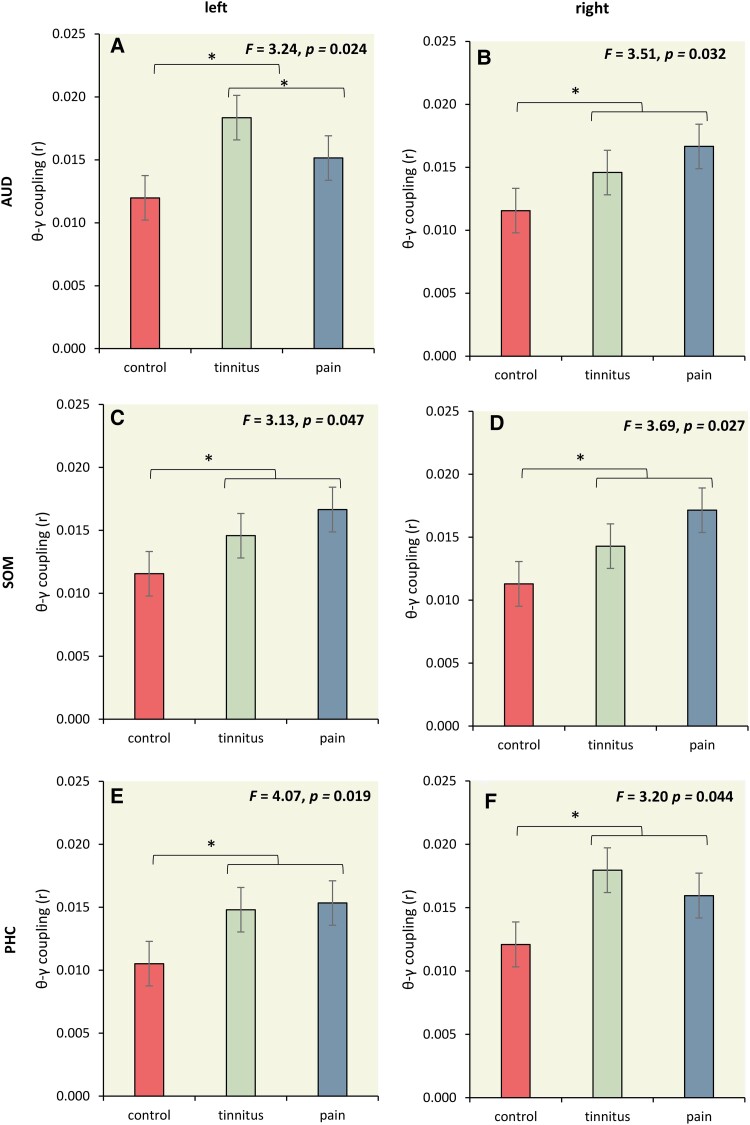
**Theta–Gamma phase-amplitude coupling.** (**A-F**) A significant effect in left and right auditory cortex, the left and right somatosensory cortex, and left and right parahippocampus. For all these areas, we see a similar significant trend, where both tinnitus and pain subjects showed increase theta–gamma phase-amplitude coupling in comparison to control subjects. No significant effect was obtained between pain and tinnitus subjects. (statistics: univariate ANOVA)

Overall, a MANOVA of theta–gamma phase-amplitude coupling for the left and right auditory cortex, the left and right somatosensory cortex, and left and right parahippocampus as dependent variables and group (controls, tinnitus, and pain) as independent variables—for the theta frequency band—showed an overall effect (*F* = 2.76, *P* = 0.001, *η^2^* = 0.11; see Fig. 7). A one-way ANOVA showed a significant effect in left and right auditory cortex, the left and right somatosensory cortex, and left and right parahippocampus. For all these areas, we see a similar significant trend, where both tinnitus and pain subjects showed increase theta–gamma phase-amplitude coupling in comparison to control subjects. No significant effect was identified between pain and tinnitus subjects.

## Discussion

A whole-brain analysis of current density fluctuations demonstrated that tinnitus and pain share elevated theta activity in the ventromedial prefrontal cortex/pregenual anterior cingulate cortex, extending into the dorsolateral and ventrolateral prefrontal cortex laterally, and anterior temporal area, both medially and laterally. A more detailed ROI analysis—of the pregenual anterior cingulate cortex—showed that tinnitus and pain both differ significantly in theta band activity from controls, but not from each other. The pregenual anterior cingulate cortex is involved in suppressing both the experience of pain^3,8,104-106^ and sound.^3,8,107-109^ This region is regarded, in conjunction with the dorsal lateral prefrontal cortex, as the apex of the descending pain inhibitory pathway in the somatosensory system, and the noise cancelling pathway in the auditory system. These pathways may run in parallel, from the dorsal lateral prefrontal cortex to the pregenual anterior cingulate cortex, extending to the reticular nucleus of the thalamus and from there to the periaqueductal grey for pain,^105,110,111^ and the adjacent tectal longitudinal column for the auditory system.^112-116^ The descending inhibitory system further extends to the rostroventral part of the medulla oblongata and to the dorsal horn to suppress further pain input. Analogously, the noise cancelling system connects from the tectal longitudinal column to the olivocochlear bundle, inhibiting auditory input.^117,118^

The ventromedial prefrontal cortex/pregenual anterior cingulate cortex is part of the default mode network, a self-referential network, likely involved in integrating pain and tinnitus into the self-percept.^119,120^ This same area is instrumental in hedonic processing in general.^121^ An increase in theta may however reflect a slowing of normal alpha activity, analogous to what has been detailed for thalamocortical dysrhythmia.^4^ In other words, theta reflects a ‘pathway that is asleep’.^122^ Thus, pain and tinnitus are the consequence of a deficient pain and noise inhibitory pathway, as has been proposed for both pain and tinnitus,^3,8,19,107^ increasing not only the sensory aspects of pain and tinnitus perception but also reducing hedonia. From a Bayesian perspective, the (para)hippocampal derived prediction of the expected auditory frequencies may be transmitted in theta ranges to the auditory and somatosensory cortex as a consequence of contextual predictions from the pregenual anterior cingulate cortex. In summary, the pregenual anterior cingulate cortex suppresses input from the sensory areas, by informing the parahippocampus to pull the missing information from memory. This hypothesis can be verified by looking at connectivity measures (see below). In terms of precision weighted prediction errors, a deficient inhibitory, noise cancelling role fits comfortably with the failures of sensory attenuation that are thought to underlie a whole range of neurological and psychiatric phenomena.^123-130^

For the gamma band, tinnitus and pain share activity increases in the auditory and somatosensory cortex, the dorsal anterior cingulate cortex and parahippocampus, extending in the temporo-occipital junction laterally. This suggests that the auditory bottom-up prediction error^131^ is propagated to the medial and lateral and descending pain and auditory networks.^3^ All these areas have been identified previously in chronic tinnitus and pain.^36,81,132-137^ The dorsal anterior cingulate cortex is part of the medial system and reflects the affective-motivational component of the pain and tinnitus,^3,104^ leading to affective experience and suffering. This is associated with the sensory-discriminative aspects of the pain and tinnitus which are encoded by the lateral system, i.e. the somatosensory and auditory cortex.^3^ This component links the pain and tinnitus to the salience network, which encodes the behavioural relevance of the pain and tinnitus.^3,104^ The parahippocampus has been identified as processing context dependent modulation of pain and tinnitus, via its contextual memory function.^69,70,138^ It extends to the temporo-occipital junction, a component of the goal-oriented central executive network.^139,140^ A more detailed ROI analysis demonstrates that in pain not only the somatosensory cortex, but also the auditory cortex evinces more gamma-band activity, as well as the parahippocampus.

Whereas multisensory integration is a good candidate for common increased gamma in multiple sensory cortices, an alternative explanation could be that unimodal deafferentation leads to a combination of deafferentation-related gamma activity in the deafferented cortex based on thalamocortical dysrhythmia, and compensatory increased gamma-controlled gain associated with sensory sensitivity increase in the other.^141,142^

Yet, tinnitus and pain are emergent properties of hierarchical processing and distributed network activity,^1,42,143,144^ and thus measures of connectivity may differentiate patients with tinnitus from pain, as activity in the respective sensory cortex clearly does not, evidenced by patients with disorders of consciousness, who have auditory and somatosensory cortex activation without conscious percepts.^45,145-147^

Functional connectivity, which evaluates statistical co-activation between different areas^148,149^ was clearly different between pain and healthy controls, as well as between tinnitus and healthy controls. And intriguingly, analogous to what has been shown for activity, functional connectivity is very similar between tinnitus and pain. For both clinical cohorts, the pregenual anterior cingulate cortex had reduced functional connectivity with the somatosensory and auditory cortices, as well as to the parahippocampal areas. This suggests that pain and tinnitus suppression is deficient, and that the suppression is deficient for contextual memory. In other words, this functional disconnection permits the somatosensory cortex and auditory hierarchy to be dominated by prior constraints in parahippocampal memory. And this is confirmed by the fact that the parahippocampus has increased functional connectivity with both the auditory and somatosensory cortex. This, however, leads to a conundrum: if both auditory and somatosensory processing pulls information from the parahippocampus, why does not every patient with tinnitus have pain and vice versa? Pain and tinnitus are often co-morbid,^150-152^ but mostly independent clinical entities.

Effective connectivity is a measure of directed connectivity;^149^ it describes from where to where the information is passed. One way of computing effective connectivity is via Granger causality analysis. In addition, tinnitus and pain differ from healthy controls in their patterns of information flow, but analogous to what has been identified for activity and functional connectivity, tinnitus and pain also share a similar pattern of information flow in theta and gamma, with one fundamental difference between the two clinical conditions. Pain is characterized by bidirectional information flow between parahippocampus and somatosensory cortex, in contrast to tinnitus, in which information in theta frequencies flows from parahippocampus to somatosensory cortex. Tinnitus is characterized by the opposite phenomenon. The parahippocampus is bidirectionally connected in theta to the auditory cortex but unidirectionally to the somatosensory cortex.

What could be the significance of this crucial difference? The parahippocampus is the contextual sensory gate to the hippocampus, and in tinnitus and pain, contextual memory information is sent from the parahippocampus to both cortices to inform both cortices on what to expect; i.e. as top-down predictions of sensory input. Yet, in deafferentation, the thalamus slows down from alpha to theta activity and sends information to the parahippocampus that it is deprived of the deafferented (i.e. missing) information, requiring the missing information so multisensory congruence is achieved, fulfilling the duck test. This leads to a vicious circle of parahippocampal-somatosensory theta effective connectivity in pain, and a vicious circle of parahippocampal-auditory theta effective connectivity in tinnitus. From a Bayesian point of view, the parahippocampus keeps sending a prediction to the auditory cortex, and the auditory cortex returns a prediction error signal, stating no bottom-up auditory input has arrived. Consequently, the parahippocampus generates the missing auditory input, which is perceived as tinnitus. This is exactly consistent with the predictive coding explanations for hallucinosis and hallucinations in computational psychiatry; namely an ‘arms race’ between the precision afforded prediction errors at sensory levels of the hierarchy and prior precision at higher levels.^127,153-155^ This vicious circle has been attributed to a primary failure of sensory attenuation in hierarchical predictive coding, that is compensated for by an increase in prior precision.^126,156^ This may represent the common mechanism for chronic tinnitus and pain^157-159^ that is expressed in a domain specific fashion in the respective sensorimotor cortices with a common involvement of the domain general, multisensory representations in the parahippocampal formation.

Yet, this leads to another conundrum. How does the theta effective connectivity link to the gamma-band-related prediction errors? It has been shown that gamma-band activity is commonly nested on theta activity, in which the theta band acts as a kind of carrier wave, and the gamma band as fast belief updating nested on the theta band.^90,100,160-162^ This nesting can be evinced by cross-frequency coupling, in which the phase of the theta is correlated with the envelope of the gamma amplitude. And indeed, as expected, the theta–gamma cross-frequency coupling is increased in pain and tinnitus in the auditory, somatosensory, and parahippocampal cortex. Also, here there is no differentiation between tinnitus and pain. This Bayesian explanation argues that the forward theta connectivity constitutes a prediction error signal (reflected by gamma activity nested on theta), which is generated by the incongruence between the backward prediction signal that maintains the tinnitus percept. Alternatively, the increased forward theta connectivity could indicate that sensory cortex exerts a greater influence of spontaneous activity in the deprived sensory cortex attempting to update (para)hippocampal predictions.

Although our research is very promising, a potential weakness is the number of different analyses. However, every method and analysis was built on the previous analysis and can be seen as a cross-validation. For example, using functional connectivity was further confirmed by effective connectivity and added additional information. However, this does not exclude that further replication needs to take place by other groups to confirm our findings.

In conclusion, this analysis offers a unique explanation of the difference between tinnitus and pain, both consequences of deafferentation. The brain seems to use universal mechanisms, irrespective of the sensory domain, to solve this sensory uncertainty, by pulling the missing information from contextual memory as a Bayesian attempt to fill in the missing information.^3,19,36^ The phenomenological difference between an auditory and somatosensory illusory or phantom percept seems to be determined not by activity, nor by functional connectivity, but by a vicious circle of constant belief updating in the absence of sensory constraints. This opens the possibility of arresting the hippocampal-sensory vicious circle through highly specific targeted neuromodulation, disrupting theta effective connectivity.

## Abbreviations

ANOVA =: analysis of covariance
AUD =: auditory cortex
MANOVA =: multivariate analysis of covariance
pgACC =: pregenual anterior cingulate cortex
PHC =: parahippocampus
ROI =: regions of interest
sLORETA =: standardized low-resolution brain electromagnetic tomography
SOM =: somatosensory cortex
